# Editorial: Physiological and molecular perspectives of stress tolerance in vegetables

**DOI:** 10.3389/fpls.2022.1004093

**Published:** 2022-09-05

**Authors:** Mostafa Abdelrahman, Lam-Son Phan Tran, Masayoshi Shigyo

**Affiliations:** ^1^Molecular Biotechnology Program, Faculty of Science, Galala University, Suez, Egypt; ^2^Department of Botany, Faculty of Science, Aswan University, Aswan, Egypt; ^3^Institute of Genomics for Crop Abiotic Stress Tolerance, Texas Tech University, Lubbock, TX, United States; ^4^Laboratory of Vegetable Crop Science, College of Agriculture, Graduate School of Sciences and Technology for Innovation, Yamaguchi University Yamaguchi City, Yamaguchi, Japan

**Keywords:** vegetable, physiological, molecular, abiotic and biotic stress, yield

## Introduction

In 2021, the Intergovernmental Panel on Climate Change (IPCC, 2021) released a recent report on the anthropogenic effects of current climate changes. Climate changes such as persistent drought, increased soil salinity and frequent heat-waves, and reductions in the quantity and quality of water resources pose serious threats to food security for the coming generations, both from a qualitative and quantitative viewpoint (Abdelrahman et al., 2020a,b,c, 2021). For these reasons, the development of climate-resilient crops will play a significant part in revolutionizing farming systems to cope with the projected extreme environmental fluctuations (Schiermeier, 2018; Abdelrahman et al., 2019). To overcome these changes, crops have developed complex mechanisms for stress tolerance, including stress perception, signal transduction, transcriptional activation of stress-responsive target genes, synthesis of enzymatic and non-enzymatic antioxidants, and production of osmoprotectants (Gupta and Huang, 2014; Resende et al., 2020). Emerging technologies from multiple research areas including plant genomics, crop breeding, plant physiology, omics-based techniques, and bioinformatics, present opportunities to improve the efficiency of screening useful agronomic traits that can enhance abiotic stress tolerance in vegetable crops. These interests have prompted us to edit this Research Topic, collecting a total of 12 contributions (six reviews and six original research articles) which cover different Physiological and Molecular Perspectives of Stress Tolerance in Vegetables. In particular, the topics cover both abiotic and biotic stress tolerance/resistance, as well as the potential molecular mechanisms involved. A discussion of these articles is given below.

## Key remarks

The physiological and biochemical levels of two different melon (*Cucumis melo*) cultivars were evaluated in response to control, drought, or salt stress conditions (Chevilly et al.). Authors reported distinctive traits for salt tolerance in melon, including phenylalanine, histidine, proline, and the Na^+^/K^+^ ratio. On the other hand, the characteristic traits for drought tolerance were the hydric potential, isoleucine, glycine, phenylalanine, tryptophan, serine, and asparagine (Chevilly et al.). These obtained results can be useful markers for breeding strategies or to predict which varieties are likely to perform better under drought or salt stress. In another study, Wang et al., functionally characterized the potential role of pumpkin *Regulator of chromosome condensation 1* (*CmRCC1*) gene involved in cold tolerance. Cold stress is the main limiting factor of cucurbit crop cultivation as it affects crop yield and quality; thus, identification of stress responsive genes is a crucial aspect of pumpkin rootstock breeding. Results indicated that *CmRCC1* overexpression in tobacco increased the gravitropic set-point angle in lateral roots, as well as root volume and diameter under cold stress. In addition, *CmRCC1* overexpression maintained photosynthetic activity under cold stress. Thus, this study highlights the positive regulatory role of *CmRCC1* in root architecture, which can be utilized in the future for improving crop yield and quality under cold stress. Song et al. investigated the relationship between antioxidant capacity in leaves and storage properties in different sweet potato (*Pomoea batatas*) cultivars, demonstrating that cultivar ‘Xu 32', which showed the best storage property, had higher antioxidant enzyme activity and lower lipoxygenase and malondialdehyde (MDA) contents. The above results revealed that storage property is highly correlated with antioxidant capacity in sweet potato leaves and negatively correlated with α-amylase activity in tuberous roots, which provides a convenient means for the screening of storage-tolerant sweet potato cultivars (Song et al.). In another study, Yi et al. investigated the biological function of radish Aquaporins (*Raphanus sativus, RsAQPs*) genes under salt stress conditions. Results indicated that seven *RsAQP* genes, such as *RsPIP1*-*3, 1*-*6, 2*-*1, 2*-*6, 2*-*10, 2*-*13*, and *2*-*14*, exhibited significant upregulation in roots of salt-tolerant radish genotype (Yi et al.). In addition, the overexpression of *RsPIP2*-*6* enhanced salt tolerance in transgenic radish hairy roots, which was evident by improved growth of transgenic radish under salt stress condition compared with wild-type (WT) plants (Yi et al.). With respect to cluster bean (*Cyamopsis tetragonoloba* L.) drought stress tolerance, RNA-seq analysis of drought-stressed vs. well-watered cluster beans revealed the crucial role of increased wax deposits on the leaf surface in combating drought stress in cluster beans under drought stress condition (Reddy et al.). Thus, further investigation about wax regulatory genes could be important for improving crop drought stress. Khandagale et al. explored the transcriptomic changes in onion (*Allium cepa*) response to *Alternaria porri*, revealing distinctive upregulation of *GABA transporter1, ankyrin repeat domain-containing protein, Xyloglucan endotransglucosylase*/*hydrolase*, and *Pathogenesis*-*related protein 5* in resistant onion genotype. Transcriptome profiling of onion response to *Alternaria porri* infection will serve as an important resource for future studies to elucidate the molecular mechanism of onion-*A. porri* interaction and to improve disease resistance in onion. Several review articles in this Research Topic summarized and discussed the recent developments in crop stress tolerance. For example, Kang et al. ummarized and discussed heat stress-responsive genes including those encoding heat shock factors and heat shock proteins, and their functional roles in heat stress tolerance of vegetable crops. Likewise, Hoshikawa et al., investigated the molecular mechanisms involved in heat stress tolerance and the challenges of developing heat-tolerant tomato varieties. Parvathi et al. discussed the progress made in deciphering the multifactorial stress responses of cucurbits and their multifactorial stress-specific traits/mechanisms/pathways and their crosstalk associated traits, both individually and in combination.

## Conclusions and future prospects

This special edition brought together interesting studies that reveal the importance of understanding molecular and physiological mechanisms in vegetable crops' response to environmental stresses. Integrated metabolome and transcriptome analysis will be essential components to decipher stress tolerance mechanisms and to identify stress-specific markers that can be utilized in breeding programs to increase yield and productivity under current and future climatic conditions. Although much is known about how plants acclimate to individual stress, little is known about how they respond to a combination of many stress factors simultaneously. Thus, future studies addressing the impact of multifactorial stress combination associated with climate changes is needed to understand how such stress combination is affecting crops. In addition, a proteomic approach has been found to be very important as it helps plant physiologists to understand what is going on in the cell due to an external stimulus. Thus, future studies using proteomics will gain much attention and might provide novel and important information for developing stress-resilient crops.

## Author contributions

All authors listed have made a substantial, direct, and intellectual contribution to the work and approved it for publication.

## Conflict of interest

The authors declare that the research was conducted in the absence of any commercial or financial relationships that could be construed as a potential conflict of interest.

## Publisher's note

All claims expressed in this article are solely those of the authors and do not necessarily represent those of their affiliated organizations, or those of the publisher, the editors and the reviewers. Any product that may be evaluated in this article, or claim that may be made by its manufacturer, is not guaranteed or endorsed by the publisher.
